# A Contemporary Systematic Approach to Assessing the Patient with Heart Failure with Reduced Ejection Fraction: Multimodal Noninvasive and Invasive Evaluation

**DOI:** 10.1155/2019/3039740

**Published:** 2019-09-11

**Authors:** Siu-Hin Wan, Paul M. McKie, John P. Bois

**Affiliations:** Department of Cardiovascular Diseases, Mayo Clinic, Rochester, MN, USA

## Abstract

Heart failure with reduced ejection fraction (HFrEF) is a progressive clinical syndrome commonly associated with left ventricle dilatation and characterized by reduced cardiac output, secondary pulmonary and systemic venous congestion, and inadequate peripheral oxygen delivery. It is common yet complex and requires synthesis of evidence-based guidelines along with strong clinical acumen. The following is a review of an illustrative case that highlights the important clinical considerations in diagnosis, assessment, and management of HFrEF commonly encountered in practice. Explanations provided highlight of the relevant pathophysiology of HFrEF as well as detailed explanations of interpretation of examinations and both noninvasive and invasive assessment in heart failure. The example provided would hopefully serve as a potential point of reference for trainees as well as healthcare practitioners for patients with HFrEF.

## 1. Introduction and Disease Overview

### 1.1. Prevalence and Etiology

5.1 million Americans suffer from heart failure with annual associated healthcare expenditures nearing $30 billion [1]. Heart failure severity is classified into stages A through D by the American College of Cardiology and the American Heart Association. Heart failure with reduced ejection fraction (HFrEF) is a clinical syndrome defined by systolic dysfunction with inadequate tissue perfusion and generally associated with an ejection fraction of less than or equal to 40%. Approximately half of the patients with heart failure have HFrEF and half have heart failure with preserved ejection fraction (HFpEF). The differential diagnosis for etiologies of HFrEF is broad (Table 1) with coronary artery disease and hypertension as the most common precipitants.

### 1.2. Pathophysiology

HFrEF arises from cardiac myocyte dysfunction leading to decreased contractile force. Neurohumoral systems such as the renin-angiotensin aldosterone and sympathetic nervous system compensate by increasing preload and heart rate. The resultant ventricular dilatation serves to maintain stroke volume and ultimately ensure sufficient systemic perfusion. However, chronic activation of these neurohumoral systems predisposes surviving myocytes to pathologic remodeling and further cardiac dysfunction [2]. In early stages of heart failure, the natriuretic peptide system counteracts the deleterious effects of maladaptive neurohumoral activation but is dysfunctional in later stages of heart failure. Ultimately, the heart reaches its limit to compensate and cardiac output is unable to meet demand.

## 2. Clinical Presentation

### 2.1. History

A seventy-three-year-old African American man with a history of long-standing paroxysmal atrial fibrillation on warfarin therapy, hypertension, dyslipidemia, and stage III chronic kidney disease (GFR 52 mL/min) presented with a two-year history of progressive dyspnea on exertion. The patient's symptoms initially occurred after walking two miles but now developed after only walking a half-mile. He denied cough, fever, orthopnea, paroxysmal nocturnal dyspnea, or angina. He noted trace bilateral lower extremity edema and occasional palpitations.

He had no known history of coronary artery or structural heart disease. There were no underlying pulmonary disorders and he was a never smoker. There was no family history of cardiovascular disease, and the patient had maintained a healthy body weight and exercise regimen, although now with decreasing tolerance.

Pertinent cardiovascular medications included warfarin for antithrombotic prophylaxis, diltiazem to maintain heart rate control, and hydrochlorothiazide for blood pressure management.

### 2.2. History Discussion

The initial differential for this case is broad and should include pulmonary pathology, deconditioning, and various cardiac etiologies.

Possible cardiac etiologies include arrhythmias, structural heart disease, and coronary artery disease. Heart failure should be considered as the clinician formulates the diagnostic approach. The clinical history is notable for bilateral lower extreme edema coupled with exertional dyspnea which is suggestive of heart failure. The clinical history is negative for orthopnea and paroxysmal nocturnal dyspnea, two specific findings in heart failure at 81% and 80%, respectively. However, with a sensitivity ranging from 20% to 30%, the lack of orthopnea or paroxysmal dyspnea does not eliminate the possibility of heart failure [3]. Finally, given the patient's age, gender, dyslipidemia, and chronic kidney disease, coronary artery disease should be considered in the primary differential with dyspnea on exertion as a possible anginal equivalent. The clinician should note that renal disease among African-Americans nearly doubles the risk of coronary artery disease.

Pertinent aspects in the history such as maintaining a healthy body weight and appropriate levels of physical activity lessen the probability that simple deconditioning is the culprit for the patient's presentation. Furthermore, the lack of smoking coupled with no prior known pulmonary disease decreases the likelihood that a primary pulmonary disorder is responsible for the patient's dyspnea on exertion.

### 2.3. Physical Examination

Upon initial presentation, the patient appeared comfortable and well nourished. The heart rate was 77 beats per minute and regular, blood pressure was 125/82 mmHg, respiratory rate was 12 breaths per minute with an oxygen saturation of 96% on room air, and temperature was 36.9 degrees Celsius. Body mass index was recorded at 25.85 kg/m^2^.

On inspection, there was jugular venous distention to the angle of the mandible (∼12 cm H_2_O). Palpation revealed that the cardiac apex was both enlarged and displaced lateral to the midaxillary line. There was no parasternal heave, and the second heart sound was not palpable. Cardiac auscultation noted a normal first and an accentuated second heart sound with wide splitting that increased during inspiration but was present throughout the respiratory cycle. A grade II/VI holosystolic murmur was noted at the apex. The murmur did not radiate nor did it change in intensity with respiration or provocative measures such as Valsalva or handgrip maneuver.

The abdominal examination revealed no evidence of hepatomegaly or ascites. The liver was nonpulsatile, and a hepatojugular reflux could not be elicited.

Examination of the extremities revealed no evidence of clubbing, sclerodactyly, joint inflammation, or rash. Grade II pitting edema to just below the knees bilaterally was noted. Posterior tibial and dorsalis pedis pulses were easily palpable bilaterally. Cutaneous and joint examination was unremarkable without evidence of rash, lesions, or joint swelling.

Pulmonary examination revealed no evidence of dullness to percussion, and the lung fields were clear without any detection of rales or decreased breath sounds.

No lymphadenopathy was appreciated upon palpation. Oral examination revealed moist mucous membranes without any other abnormalities.

### 2.4. Physical Examination Interpretation

There are several key elements in the physical examination that aid in focusing the differential diagnosis. Most importantly, the cardiac examination suggests a dilated cardiomyopathy given the findings of an enlarged and displaced cardiac apex.

The prominent second heart sound coupled with persistent splitting suggests pulmonary hypertension. The loud S2 in pulmonary hypertension is generated by the increased pulmonary pressure closing the pulmonic valve. There is persistent splitting of the second heart sound that increases with inspiration. Increased pulmonary pressure prolongs the right ventricular ejection time, which delays closure of the pulmonic valve resulting in increased splitting of the second heart sound. The increased return of venous blood to the right ventricle during inspiration further increases the right ventricular ejection time, leading to the finding of wide persistent splitting. Persistent splitting may also be found in other conditions that delay emptying of the right ventricle during systole such as right bundle branch block.

The location, timing, and nature of the grade II/VI holosystolic murmur at the apex are consistent with mitral regurgitation. Mitral regurgitant murmurs are typically appreciated best at the cardiac apex and are often persistent throughout systole. In severe mitral regurgitation, the murmur will increase with increased afterload and will decrease with decreased preload. However, this murmur did neither and is soft in its intensity, suggesting mild or mild to moderate regurgitation.

A proposed mechanism for the mitral regurgitant murmur based upon the physical examination findings is dilation of the mitral valve annulus with resultant decreased ability of the mitral valve leaflets to coapt resulting in secondary mitral regurgitation. An abnormality with the valve itself known as primary mitral regurgitation can be seen in such diseases as mitral valve prolapse. However, a murmur of mitral valve prolapse will often include an audible systolic click, corresponding with prolapse of the valve followed by a murmur. Furthermore, provocative maneuvers that increase afterload or decrease preload should accentuate the murmur of mitral valve prolapse, neither of which was appreciated in this case.

With the suspicion of dilated cardiomyopathy as well as pulmonary hypertension, the next task is to determine if other examination findings suggest the degree of the patient's physiologic compensation. The jugular venous pulse was distended and 2+ peripheral edema was appreciated, suggesting moderate intravascular volume overload. While the lung fields were clear upon auscultation, this finding can be less sensitive in determining clinical volume status in chronic heart failure patients given the ability of the pulmonary lymphatic system to engorge over time and accommodate excess fluid.

There is little on examination to suggest a systemic cause of the suspected dilated cardiomyopathy. For instance, there was no macroglossia that would be appreciated in amyloidosis, no mention of hypogonadism that is associated with hemochromatosis, and no cutaneous abnormalities such as erythema nodosum which is noted in up to a quarter of patients with sarcoidosis.

In regard to the suspicion of pulmonary hypertension, there are several key examination findings that reveal the status of the right ventricle as well as possible clues as to an underlying etiology. First, the patient had appropriate oxygen saturation on room air and no evidence of peripheral cyanosis. Next, it was noted on the examination that a parasternal heave was not appreciated. While the presence of a right ventricular heave is specific for right ventricular hypertrophy (95.7%), it is not sensitive (37.5%) and therefore right ventricular hypertrophy cannot be ruled out by the absence of this finding [4]. Similarly, the hepatojugular reflux, which was not appreciated on examination, is specific (100%) but not particularly sensitive (66%) for diagnosing right ventricular heart failure [5]. The examination includes many pertinent negatives as to possible etiologies of pulmonary hypertension. For instance, kyphosis was noted, but this was mild and therefore unlikely to be causing restrictive lung disease. Auscultation of the lung fields did not reveal any rales that would be associated with interstitial lung disease nor was there evidence of peripheral clubbing. The patient does not have evidence of joint inflammation or skin rash that would suggest an underlying autoimmune disease that would result in pulmonary hypertension. A significant pertinent positive is the discovery of a dilated left ventricle raising the suspicion of an elevated left ventricular end-diastolic pressure contributing to increased pressures in the pulmonary vasculature (Table 2).

### 2.5. Case Presentation Summary

A seventy-three-year-old male with a history of paroxysmal atrial fibrillation, dyslipidemia, and chronic kidney disease stage III presented with progressive exertional dyspnea. The differential was broad and included pulmonary disorders such as restrictive or obstructive lung disease, deconditioning, and a variety of cardiac conditions such as coronary artery disease, heart failure, or sequelae from atrial fibrillation such as poor heart rate control.

The clinical examination refined this differential with findings suggestive of a dilated cardiomyopathy with mild to moderate secondary mitral regurgitation and pulmonary hypertension. The subsequent noninvasive evaluation should focus upon confirming these clinical suspicions and defining specific etiologies to guide therapeutic strategies.

### 2.6. Noninvasive Assessment

Laboratory evaluation was as follows (findings outside of the normal limits bolded):

  Hemoglobin: 13.3 (13.5–17.5 g/dL)
   Leukocytes: 9.6 × 10^9^ (3.5–10.5 × 10^9^/L)
  Mean corpuscular volume: 83 (81.2–95.1 fL)
   Platelet count: 204 × 10^9^ (150–450 × 10^9^/L)
  Creatinine: 1.6 (0.8–1.3 mg/dL)
   Sodium: 143 (135–145 mmol/L)
   Blood urea nitrogen (BUN): 29 (8–24 mg/dL)
    Potassium: 4.2 (3.6–5.2 mmol/L)
  Alkaline phosphatase: 125 (45–115 U/L)
    Aspartate aminotransferase (AST): 29 (8–48 U/L)
   Uric acid: 25 (3.7–8.0 mmol/L)
    Thyroid-stimulating hormone (TSH): 2.0 (0.3–5.0 MIU/L)
   Amino-terminal proB-type natriuretic peptide (NT-proBNP): 9245 (<107 pg/mL)
    Total iron: 114 (50–150 mcg/dL)
    Total iron-binding capacity (TIBC): 287 (250–400 mcg/dL)
    Ferritin: 112 (24–336 ng/ml)
    Total cholesterol: 208 (<200 mg/dL)
    Triglycerides: 71 (<150 mg/dL)
    High-density lipoprotein (HDL): 59 (>40 mg/dL)
    Low-density lipoprotein (LDL): 125 (<130 mg/dL)
    Serum and urine protein electrophoresis (SPEP and UPEP) negative for monoclonal gammopathy

**Figure 1:**
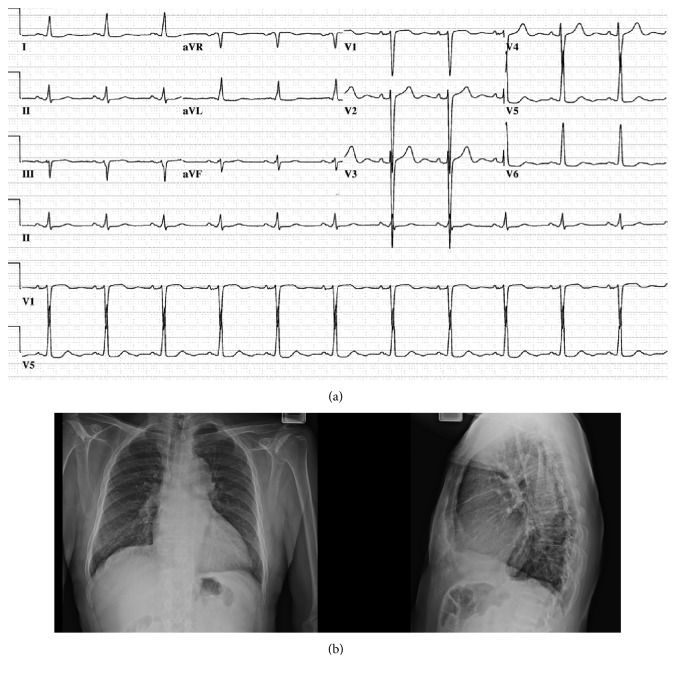
(a) 12-Lead electrocardiogram: ECG demonstrating sinus rhythm with left ventricular hypertrophy based upon Cornell voltage criteria. (b) Anteroposterior and lateral chest x-ray: demonstrating mild cardiomegaly, a tortuous aorta, and mild interstitial prominence greatest at the right lung base.

A twelve-lead resting ECG demonstrated a normal sinus rhythm, left ventricular hypertrophy, and nonspecific ST changes (Figure 1(a)). A twenty-four-hour ambulatory blood pressure monitor documented an average blood pressure of 139/82 mmHg with a maximum blood pressure of 180/118 mmHg. A twelve-lead twenty-four-hour ambulatory Holter monitor noted sinus rhythm with a heart rate varying between 53 and 123 beats per minute with an average heart rate of 70 beats per minute. Two three- to four-beat runs of ventricular tachycardia were noted with a peak rate of 132 beats per minute. No other premature ventricular or supraventricular contractions were noted and the patient remained asymptomatic during the Holter evaluation.

A posteroanterior and lateral chest x-ray noted mild cardiomegaly, a tortuous aorta, and a mild interstitial prominence greatest at the right lung base (Figure 1(b)).

**Figure 2:**
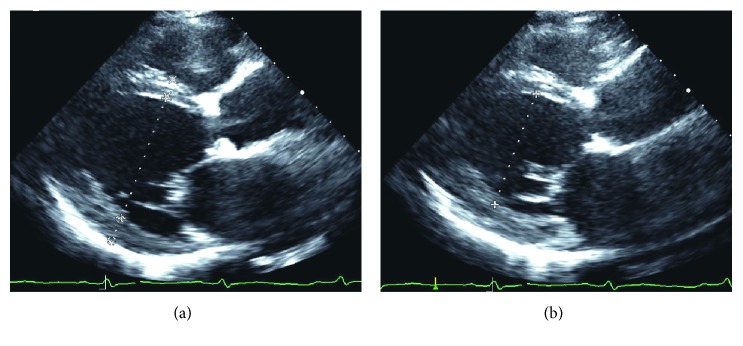
Transthoracic echocardiogram for cardiac size and function. (a) Parasternal long-axis view demonstrating severe dilatation (end-diastolic diameter of 64 mm) with normal anterior septal and inferior lateral wall thickness (9 and 12 mm, respectively). (b) Similar view demonstrating increased end-systolic dimension (57 mm) consistent with reduced ejection fraction.

**Figure 3:**
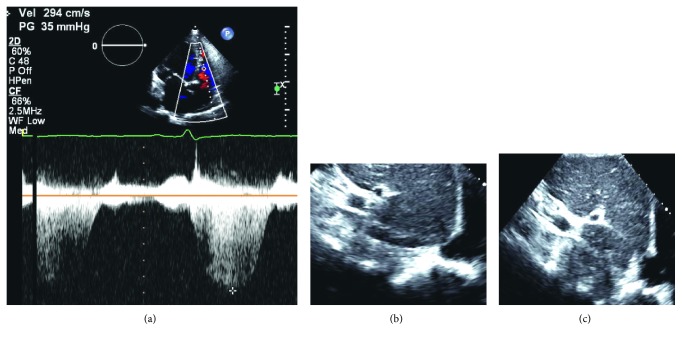
Transthoracic echocardiogram to determine right ventricular systolic pressure (RVSP). (a) Continuous-wave Doppler measurement of peak tricuspid regurgitant velocity of 2.9 m/s. Right ventricular systolic pressure is calculated via the modified Bernoulli equation Δ*P*_TV_=4 × (*V*_*TR*_)^2^ or Δ*P*_*TV*_=4 × (2.9 m/s)^2^=34 mmHg. A small inferior vena on expiration (b) that completely collapses on inspiration (c) consistent with a mean right atrial pressure of 5 mmHg.

Transthoracic echocardiography demonstrated severe left ventricular enlargement (two-dimensional end-diastolic dimension of 64 mm (expected 37–51) and end-systolic dimension of 54 mm (expected 22–34)) with an ejection fraction of 39% when calculated by biplane volumes (Figures 2(a) and 2(b)). The posterior wall thickness was measured at 12 mm, and the left ventricular mass index was 185 g/m^2^. Generalized left ventricular hypokinesis was appreciated. Mild-moderate central mitral valve regurgitation was noted (effective regurgitant orifice of 0.16 cm^2^ with a regurgitant volume of 34 cc by proximal isovelocity surface area (PISA)) and was likely secondary to mitral annular dilation. Elevated left ventricular filling pressure was reported with an E to A ratio of 0.67 and a medial E to e' ratio of 20. In restrictive physiology, with elevated left atrial pressures, early diastolic filling is expected to occur rapidly once the mitral valve opens, thus leading to a steep deceleration time. Right ventricular assessment noted normal right ventricular size and function with an estimated right ventricular systolic blood pressure of 39 mmHg based upon a peak tricuspid regurgitant velocity of 2.90 m/sec and an inferior vena cava that was normal in size and collapsed greater than fifty percent of its diameter with inspiration (Figures 3(a)–3(c)).

### 2.7. Noninvasive Diagnostic Discussion

The laboratory evaluation is notable for an elevated NT-proBNP which is most commonly elevated in HF, although elevation can be seen in any disease states with increased myocyte stretch or inflammation. The history of chronic kidney disease is confirmed with a calculated GFR of 52 mL/min, consistent with stage III disease. Importantly, TSH, serum ferritin, and monoclonal protein studies are within normal limits, suggesting that thyroid abnormalities, hemochromatosis, or amyloidosis are not playing a role in the patient's clinical presentation. However, if there were specific findings on clinical review, such as neuropathy and macroglossia, or echocardiographic features such as concentric hypertrophy that would be concerning for possible amyloidosis, then tissue acquisition would be recommended for pathologic diagnosis.

The ECG suggests left ventricular hypertrophy (LVH) based upon the Cornell voltage criteria. This criterion sums the R wave in aVL and the S wave in V3, and if greater than 20 mm in females or 28 mm in males, suggests LVH.

**Figure 4:**
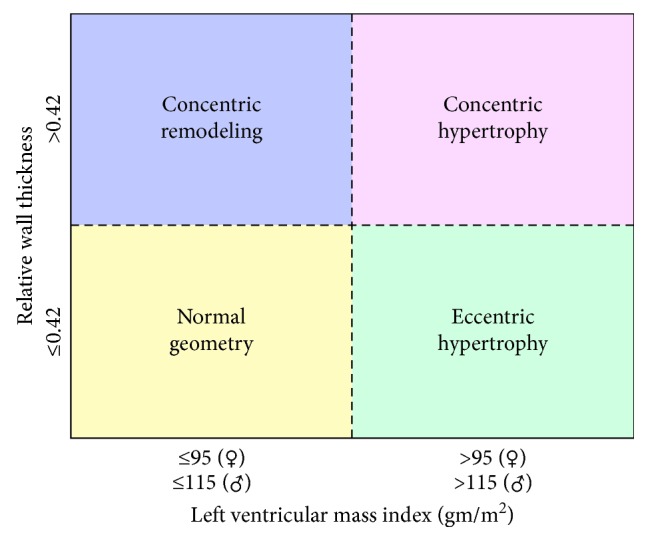
Characterization of left ventricular hypertrophy. The patient's ECG (Figure 2) suggests left ventricular hypertrophy. Transthoracic echocardiographic measurement of relative wall thickness (relative  wall  thickness=(2 × posterior  wall  thickness)/(LVEDD)) coupled with left ventricular mass index can determine the specific type of hypertrophy. In the current case, the patient's eccentric hypertrophy is consistent with volume overload secondary to a dilated cardiomyopathy (image from Lang RM. JASE. 2005; 18: 1440–1463).

The echocardiogram measurements can be utilized to further characterize the patient's hypertrophy (Figure 4). First relative wall thickness can be measured by the following equation (where LVEDD is equal to left ventricular end-diastolic dimension):

(1) $$\begin{array}{c} \text{relative wall thickness}=\frac{\left(2\times \text{posterior wall thickness}\right)}{\left(\text{LVEDD}\right)}. \end{array}$$

In the current case,

(2) $$\begin{array}{c} \text{relative wall thickness}=\frac{\left(2\times 12\text{ mm}\right)}{64\text{ mm}}=0.375. \end{array}$$

Next, a relative wall thickness of 0.375 can be combined with a calculated left ventricular mass index by echocardiogram of 185 g/m^2^ to suggest eccentric hypertrophy (Figure 4). Studies suggest that specification of type of hypertrophy not only correlates with underlying pathophysiology (concentric hypertrophy associated with pressure overload and eccentric hypertrophy associated with volume overload) but also has prognostic implications [6].

**Figure 5:**
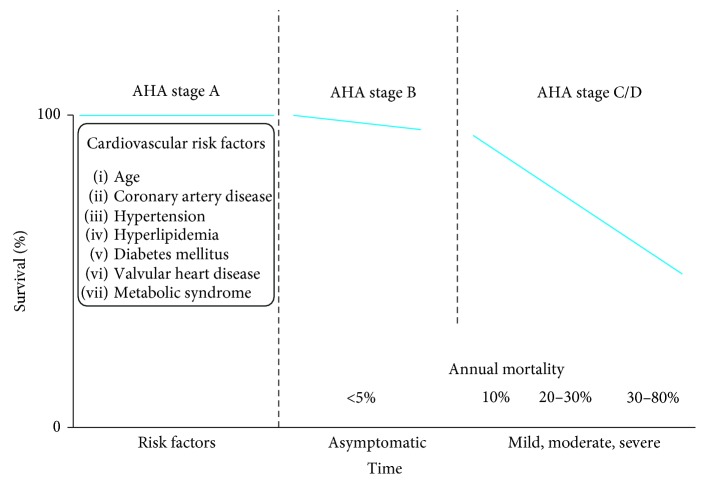
Correlation of AHA stage and survival. Note the marked deterioration in survival once the patient becomes symptomatic (the patient in the current case is AHA class C, mild to moderate with an annual mortality between ten and twenty percent) (adapted from Mayo Board Review, Figure 93.1 (page 859)).

Echocardiographic findings are most consistent with a dilated cardiomyopathy given the global hypokinesis and no 2D evidence of infiltrative disorders such as amyloidosis or sarcoidosis. The global hypokinesis suggests a nonischemic etiology, but ischemic heart disease cannot be ruled out. The finding of a depressed ejection fraction with the presentation of dyspnea confirms HFrEF as the diagnosis. The patient is classified as AHA stage C based on reduced ejection fraction and symptoms (Figure 5).

Echocardiographic assessment of diastolic function suggests increased filling pressures. The Doppler E wave, or early diastolic filling of the left ventricle, reflects the pressure gradient between the left atrium and the left ventricle [7], whereas the tissue Doppler e' depicts the rate of myocardial relaxation [8]. The elevated E/e' ratio indicates high filling pressures with impaired myocardial tissue relaxation corresponding with a pulmonary capillary wedge pressure greater than 12 mmHg [9]. An elevated E/e' (>15) is a sensitive but not specific determinant for left ventricular filling pressures [8]. In normal physiologic states, lateral e' is greater than medial e'. According to the 2016 ASE Diastolic Function Guidelines, the average of the lateral e' and medial e' should be used in determining left ventricular filling pressures in the setting of reduced ejection fraction.

**Figure 6:**
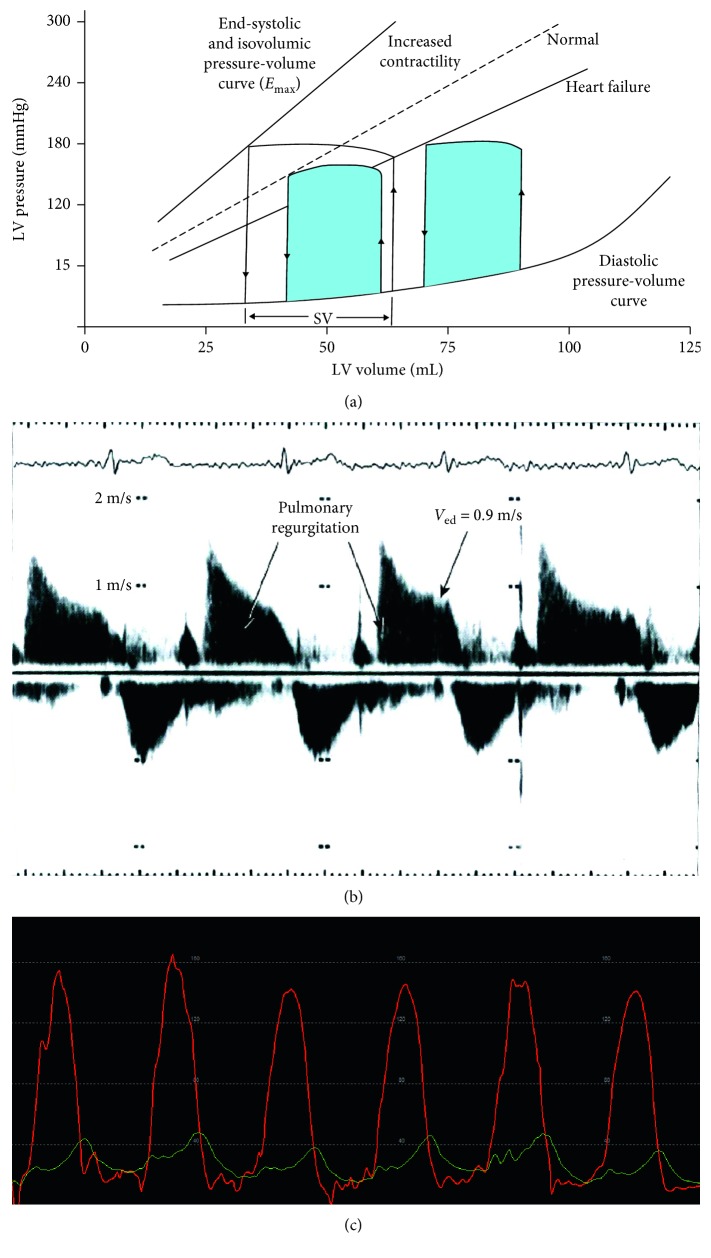
(a) Pathophysiology of dilated cardiomyopathy. The left ventricle reacts to an initial insult that decreases contractile force by increasing the end-diastolic volume (*x*-axis). Initially, left ventricular end-diastolic pressure (*y*-axis) remains low (<15 mmHg) and the patient remains asymptomatic (AHA class B). However, if progressive dilatation occurs, the end-diastolic pressure begins to rise with resultant dyspnea (AHA class C). Further deterioration leads to a reduction in stroke volume and inability to maintain necessary end-organ perfusion (AHA class D). (a) is found in Mayo Board Review as Figure 91.7 (page 849). (b) Transthoracic echocardiogram to determine RVEDP. Typically obtained in the parasternal short-axis view at the cardiac base, continuous-wave Doppler of the pulmonary regurgitant signal coupled with right atrial pressure can estimate pulmonary artery end-diastolic pressure (PADP) via PADP=Δ*P*_PVED+RAP. (b) is found in Mayo Board Review as Figure 79.7 (page 743). (c) Left ventricular (red) and pulmonary capillary wedge (PCWP) (green) tracings. Note the elevated left ventricular end-diastolic pressure (white arrow), elevated PCWP (green tracing) with prominent “v” wave (blue arrow).

The underlying pathophysiologic mechanism for HFrEF and associated LV dilatation is illustrated in Figure 6(a). An initial insult decreases left ventricular contractile force by increasing the EDV (*x*-axis). Initially, left ventricular end-diastolic pressure (LVEDP) (*y*-axis) remains low (<15 mmHg) and the patient remains asymptomatic (AHA class B, Figure 5). However, if progressive dilatation occurs, the LVEDP begins to rise with resultant dyspnea (AHA class C). Further deterioration leads to a reduction in stroke volume and inability to maintain necessary end-organ perfusion (AHA class D).

The chest x-ray is consistent with left ventricular and not right ventricular enlargement which is concordant with the physical examination findings. There is no evidence of lymphadenopathy that would be associated with sarcoidosis.

The ambulatory blood pressure and Holter monitors yield important data. Notably, the patient has suboptimal blood pressure control but does have appropriate rate control. Therefore, it is unlikely that he is suffering from a tachycardic or premature ventricular contraction-induced cardiomyopathy. However, his afterload is significantly elevated, particularly given his reduced ejection fraction, which may be playing a role in his symptomatology.

As indicated by the physical examination, the systolic murmur was consistent with secondary mitral regurgitation due to mitral annular dilatation. On echocardiography, a large central jet of regurgitation associated with central lack of coaptation in the setting of left ventricular and left atrial dilatation would support secondary mitral regurgitation. The right ventricular size and function are within normal limits which are consistent with the physical examination findings of no parasternal lift, jugular venous pressure elevation, ascites or hepatojugular reflux, and minimal peripheral edema. Interestingly, the right ventricular systolic pressure is only mildly elevated at 39 mmHg (normal at rest 30 mmHg and 40 mmHg with exertion).

### 2.8. Potential “Discrepant” Measurements

The right ventricular systolic pressure as measured by noninvasive Doppler echocardiography appears mildly elevated in this patient and is discordant with the physical examination findings of a pronounced and persistently split second heart sound throughout the precordium as well as deteriorating exercise tolerance.

Doppler echocardiography assesses right ventricular systolic pressure (RVSP) by employing the modified Bernoulli equation, which correlates pressure change to velocity via the following equation:

(3) $$\begin{array}{c} \Delta P_{\text{TV}}=4\times {\left(V_{\text{TR}}\right)}^{2}, \end{array}$$

where Δ*P*_TV_ is equal to the systolic pressure gradient across the tricuspid valve in mmHg and *V*_TR_ is the Doppler assessment of peak tricuspid regurgitant velocity in m/s.

Subsequently, the size and degree of collapse of the inferior vena cava can be utilized to estimate right atrial pressure (RAP). These two values can be combined to estimate RVSP:

(4) $$\begin{array}{c} \text{RVSP}=\Delta P_{\text{TV}}+\text{RAP}. \end{array}$$

This utility of this measurement is dependent upon (1) absence of pulmonary stenosis, (2) ability to obtain and correctly measure the tricuspid regurgitant velocity signal during Doppler examination, and (3) correct estimation of the right atrial pressure. Importantly, in severe tricuspid regurgitation, Doppler assessment of RVSP might be underestimated [10]. In these specific scenarios of a discrepancy between physical examination findings and noninvasive evaluation, catheter-based assessment would yield a more accurate measurement of RVSP.

Prior studies have demonstrated that right ventricular systolic pressure by echocardiography could accurately be obtained in 87% of patients. Moreover, simultaneous assessment of right ventricular systolic pressure by invasive hemodynamics and Doppler echocardiography demonstrates excellent correlation (correlation coefficient, *R* = 0.96) [11].

In those patients where a tricuspid regurgitant signal cannot be obtained, then end-diastolic pulmonary regurgitant velocity can be measured as demonstrated in Figure 6(b) (normal less than 10 mmHg). The following equation can be utilized to calculate end-diastolic pulmonary artery pressure:

(5) $$\begin{array}{c} \text{PADP}=\Delta P_{\text{PVED}}+\text{RAP,} \end{array}$$

where PADP indicates end-diastolic pulmonary pressure, Δ*P*_PVED_ equals the end-diastolic pressure gradient across the pulmonic valve, and RAP indicates right atrial pressure.

## 3. Invasive Assessment

When is invasive evaluation indicated for HFrEF?

The initial invasive hemodynamic evaluation of the ambulatory HF patient may include the following [12]:

1. A coronary angiogram to determine the burden of coronary atherosclerosis and the possibility of ischemic cardiomyopathy as the underlying etiology for the systolic heart failure (Level IIc).
2. An endomyocardial biopsy when a “specific diagnosis is suspected that would influence therapy” (Level IIc). A routine endomyocardial biopsy should not be routinely performed in all HF patients (Level III).
3. Hemodynamic assessment when systemic or pulmonary vascular resistance is uncertain (Level IIc).

Invasive assessment to confirm elevated LVEDP and resultant type II pulmonary hypertension is a class IIb recommendation by the ACC/AHA [12] and the European Society of Cardiology [13]. One could argue in this case that the elevated E/e' on Doppler echocardiography coupled with the underlying cardiomyopathy already suggests elevated left ventricular end-diastolic filling pressures and that treatment should be initiated without the need for further refinement of the diagnosis by catheterization.

However, in the current case, invasive hemodynamic assessment will clarify both the severity and the nature of the pulmonary hypertension, predict response to drug intervention, and aid in determining prognosis. Also, angiography can exclude CAD as underlying mechanism for HF.

In regard to the severity of PH, there is a discrepancy between physical examination findings of a prominent second heart sound that is widely split and only mild elevation of right ventricular systolic pressure on Doppler echocardiography. Measurement of right ventricular systolic pressure during cardiac catheterization will aid in clarifying the severity of pulmonary hypertension. Furthermore, measurement of the wedge pressure (PCWP) and transpulmonary gradient (TPG), which will be described in further detail in the following sections, will determine which type of PH the patient suffers from (discussed in following sections). Furthermore, measurement of the severity of pulmonary hypertension by documenting the transpulmonary gradient and pulmonary vascular resistance will aid in assessing prognosis [13]. Lastly, cardiac catheterization can demonstrate whether acute afterload reduction significantly lowers the TPR which may also impact prognosis particularly in heart transplant candidates [13].

What testing should be performed at invasive cardiac catheterization?

In approaching this case, what tests would you select from the following options?

 [X] Right heart pressure assessment (right atrial (RA), right ventricular (RV), pulmonary artery (PA), and pulmonary capillary wedge (PCW) pressures)
 [X] Full saturation run (inclusive of peripheral, inferior vena cava (IVC), superior vena cava (SVC), and arterial measures)
 [X] Fick cardiac outputs
   [_] Thermodilution cardiac outputs
   [_] Left ventricular pressure assessment (retroaortic)
   [_] Left atrial (LA) pressure assessment (transseptal)
 [X] Drug study: pulmonary vs. systemic vasodilator
   [_] Intravenous fluid challenge
   [_] Exercise study
 [X] Coronary angiography
   [_] Coronary vasospasm study
   [_] Ventriculography
   [_] Double-sampling dye curves
   [_] Other maneuvers

### 3.1. Invasive Assessment Planning

There are two main objectives for the invasive assessment of this case. First, a diagnostic coronary angiogram should be performed to evaluate the presence and extent of coronary atherosclerosis. This will help to determine if the patient's dilated cardiomyopathy is ischemic in nature. After completion of the coronary angiogram and with arterial access already in place, the aorta can be crossed in a retrograde fashion in order to assess left ventricular end-diastolic pressure.

The second objective is to confirm and further define the SVR and presence of pulmonary hypertension and how they respond to drug intervention. For this portion of the study, a right heart catheterization should be performed for assessment of the right atrial, right ventricular, pulmonary artery, and pulmonary capillary wedge pressures. These assessments will allow calculation of the TPG, demonstrated below. Furthermore, during the right heart catheterization, measurement of the mixed venous blood saturation and the arterial saturation should be obtained as well as measured assessment of oxygen consumption so that the cardiac output may be calculated via the Fick equation and subsequently the pulmonary vascular resistance (PVR) determined.

If the patient's PCWP is elevated as is suspected, then the final step will be to determine the response to acute systemic arterial vasodilator therapy such as nitroprusside. The presence or absence of a decrease in pulmonary capillary wedge pressure and ultimately TPG suggests an improved prognosis particularly in heart transplant candidates.

The clinical history, physical examination, and noninvasive assessment do not suggest an etiology that would both be readily identifiable on endomyocardial biopsy and that would alter therapeutic strategy. Therefore, in accordance with ACC/AHA guidelines, an endomyocardial biopsy was not pursued [14].

### 3.2. Pitfalls to Avoid in Invasive Assessment of HF and Type II Pulmonary Hypertension

There are several critical points to consider when assessing both HF and pulmonary hypertension. The first issue to consider is appropriate method for determining cardiac output. As will be demonstrated in the following section, cardiac output is utilized to determine PVR and the degree of PVR impacts prognosis. There are several methods utilized to determine cardiac output in the invasive hemodynamics laboratory. The thermodilution method analyzes the change in blood temperature over time after a cooled injectant has been introduced to the system. This method has the potential to underestimate cardiac output in patients with markedly low output states or severe tricuspid regurgitation. If the cardiac output were to be underestimated, then the pulmonary vascular resistance would be overestimated. The Fick equation utilizes oxygen consumption to determine cardiac output (equation noted below). Oxygen consumption can be measured in the laboratory or can be “assumed” based upon body weight. However, the assumed oxygen consumption value is often inaccurate and should be avoided as its potential inaccuracy can result in a misleading PVR.

Second, the assessment of PCWP as a surrogate for left ventricular end-diastolic pressure (LVEDP) has been shown to underestimate the true LVEDP. If the operator relied solely on PCWP, then the patient could be misclassified as type I PAH when indeed they had type II PH and be initiated on a drug therapy that would be ineffective and possibly even deleterious. A prior investigation has demonstrated that 53% of cases classified as type I PH based upon a low PCWP were reclassified as type II PH when LVEDP was measured and was demonstrated to be greater than 15 mmHg [15].

Lastly, caution should be utilized when considering the appropriate agent to administer during the drug study. In type I PAH, a pulmonary vasodilator agent such as nitric oxide is administered to determine if the mean pulmonary arterial pressure can be lowered while maintaining or improving cardiac output. Pulmonary vasodilating agents increase preload, and in type II PAH where the LVEDP is already elevated, this can lead to acute volume overload resulting in pulmonary congestion. The correct agent to use in postcapillary PH (type II PH) is an afterload reducing agent such as nitroprusside. This will allow the investigator to determine if better systemic blood pressure control would alleviate the elevated LVEDP and in turn improve the patient's symptomatology.

### 3.3. Catheterization Results

Venous and arterial access was obtained via a 7-French right femoral vein sheath and a 6-French right femoral arterial sheath. Judkins left 4 and Judkins right 4 catheters were utilized to engage the left and right coronary system, respectively. Coronary angiography noted mild diffuse disease. The catheter was then advanced into the left ventricle where an end-diastolic pressure of 24 mmHg was recorded.

The catheter was withdrawn into the aorta, and the blood pressure was measured, noted below. Hemoglobin was drawn and noted to be 12.4 g/dL.

Next oxygen consumption was measured at the bedside to be 305.00 ml/min. Arterial oxygen saturation was 92%, and mixed venous blood saturation was 45%. Subsequently, a 7-French 110 cm balloon wedge pressure catheter was introduced through the femoral sheath, and the following measurements were obtained:

   Aortic blood pressure (mmHg): 144/75 (Figure 6(c))
   Right atrial pressure (mmHg): 14/11, mean = 10
   Right ventricular pressure (mmHg): 73/8, end-diastolic pressure = 16
   Pulmonary artery pressure (mmHg): 72/32, mean = 49. Oxygen saturation = 45%
   Pulmonary capillary wedge pressure (mmHg): mean = 26. Oxygen saturation = 95% (Figure 6(c))
   Cardiac output: 3.9 L
   Pulmonary vascular resistance (Woods unit, WU): 5.96

A nitroprusside drip was administered at 2 *μ*g/kg/min, and the following data were recorded:

   Aortic blood pressure (mmHg): 92/47
   Arterial oxygen saturation: 97%
   Oxygen consumption: 340.00 ml/min
   Pulmonary artery pressure (mmHg): 46/15, mean = 26. Oxygen saturation: 73%
   Pulmonary capillary wedge pressure (mmHg): 10
  Cardiac output: 8.3 L
   Pulmonary vascular resistance (Woods unit, WU): 1.5

**Figure 7:**
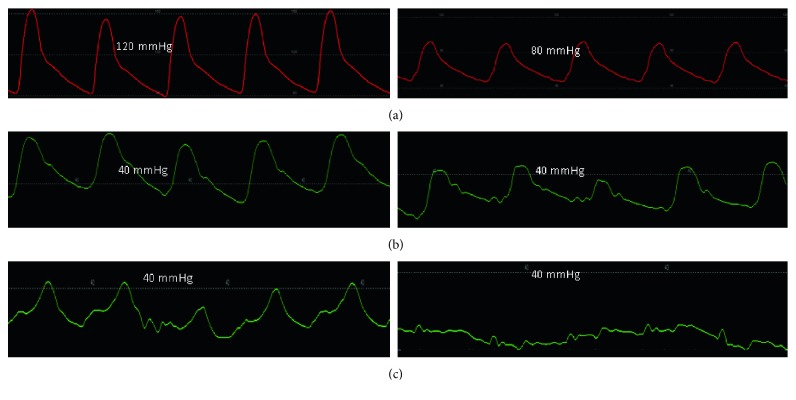
Response of systemic, pulmonary arterial, and pulmonary capillary wedge pressure to nitroprusside. The aortic blood pressure and consequently the LVEDP decreased markedly with introduction of nitroprusside (a). Subsequently, the MPAP (b) and the PCWP (c) demonstrated a marked decrease in pressure measurements.

The changes in systemic, MPAP, and PCWP are demonstrated in Figures 7(a)–7(c).

### 3.4. Catheterization Result Discussion

The coronary angiogram demonstrates no obstructive coronary disease consistent with the diagnosis of nonischemic dilated cardiomyopathy.

The measurements prior to nitroprusside administration confirm markedly elevated filling pressure and low CO consistent with HF. Type II pulmonary hypertension is also present.

To better assess the pulmonary hypertension, the transpulmonary gradient should be calculated:

(6) $$\begin{array}{c} \text{TPG}=\text{MPAP}-\text{PCWP,} \end{array}$$

where TPG is equal to the transpulmonary gradient, MPAP is mean pulmonary arterial pressure, and PCWP is pulmonary capillary wedge pressure. In our case,

(7) $$\begin{array}{c} \text{TPG}=49\text{ mmHg}-26\text{ mmHg}=23\text{ mmHg}. \end{array}$$

The TPG is ≥12 mmHg which is consistent with postcapillary reactive pulmonary hypertension whereas nonreactive postcapillary PH is ≤12 mmHg. Increased vasomotor tone with accompanying pulmonary artery remodeling underlies the pathophysiology of reactive PH [13]. It is unclear why some type II PH patients develop an elevated TPG.

Next, cardiac output should be calculated using the Fick equation as follows:

(8) $$\begin{array}{c} \text{CO}=\frac{\left({\text{VO}}_{2}\right)}{\left(\left({\text{C}}_{\text{a}}{\text{O}}_{2}-{\text{C}}_{\text{v}}{\text{O}}_{2}\right)\times 10\right)},\\ {\text{C}}_{\text{a}}{\text{O}}_{2}=\text{hemoglobin concentration}\left(\frac{\text{g}}{\text{dL}}\right)\times 1.36\text{ ml}\frac{{\text{O}}_{2}}{\text{g}}\text{hemoglobin}\times \text{arterial oxygen saturation}\left(\%\right),\\ {\text{C}}_{\text{v}}{\text{O}}_{2}=\text{hemoglobin concentration}\left(\frac{\text{g}}{\text{dL}}\right)\times 1.36\text{ ml}\frac{{\text{O}}_{2}}{\text{g}}\text{hemoglobin}\times \text{venous oxygen saturation}\left(\%\right), \end{array}$$

where CO is the cardiac output, C_a_O_2_ is the oxygen content of arterial blood, and C_v_O_2_ is the oxygen content of venous blood. Oxygen consumption is given at 305 ml/min, hemoglobin is given at 12.4 g/dL, arterial oxygen saturation was 92%, and venous oxygen saturation was 45%. Entering these data, CO can be solved for:

(9) $$\begin{array}{c} \text{ CO}=\frac{305}{\left(\left(15.5-7.6\right)\times 10\right)}=3.86\text{ L}/\text{min},\\ \text{CI}=\frac{\text{CO}}{\text{BSA}}=\frac{3.9}{1.73}=2.3\frac{\text{ L}/\text{min}}{{\text{m}}^{2}}. \end{array}$$

CO can then be utilized to determine pulmonary vascular resistance as follows:

(10) $$\begin{array}{c} \text{PVR}=\frac{\left(\text{MPAP}-\text{PCWP}\right)}{\text{CO}}. \end{array}$$

where PVR is the pulmonary vascular resistance, MPAP is the mean pulmonary artery pressure, MPCWP is the mean pulmonary capillary wedge pressure, and CO is the cardiac output. Therefore, prior to nitroprusside administration, PVR equals

(11) $$\begin{array}{c} \text{PVR}=\frac{\left(49-26\right)}{3.86}=5.9\text{ WU}. \end{array}$$

Normal PVR is ≤3 WU. This patient's elevated pulmonary vascular resistance is consistent with reactive type II PH.

Note that with the administration of nitroprusside, the systemic blood pressure fell from 144/75 mmHg to 92/47 mmHg. With this acute afterload reduction, there was a marked drop in the MPAP to 26 mmHg, the PCWP to 10 mmHg, and the PVR to 1.5 WU. These findings are reflected in the hemodynamic tracings noted in Figures 7(a)–7(c). The responsiveness of the pulmonary vascular resistance denotes reversibility, suggesting that the underlying pathophysiologic mechanism for the reactive type II PH is increased vasomotor tone rather than remodeling [13].

Also of critical importance is the more than twofold increase in CO from 3.9 to 8.3 L/min. This increase is attributable to the increased VO_2_ (from 305 ml/min to 340 ml/min) as well as the increase in mixed venous oxygen saturation from 45% to 73%. The arterial oxygen also increased from 92% to 97%. The postnitroprusside cardiac output is therefore calculated as follows:

(12) $$\begin{array}{c} \text{CO}=\frac{340}{\left(\left(16.4-12.3\right)\times 10\right)}=8.3\text{ L}/\text{min}. \end{array}$$

### 3.5. Synthesis

The left ventricular hemodynamic tracing (Figure 6(c)) is consistent with an elevated LVEDP which correlated with the transthoracic echocardiographic finding of an E to e' ratio of 20. Moreover, the “V” seen on the PCWP tracing (Figure 6(c)) is consistent with the known mitral regurgitation and elevated filling pressures. Nitroprusside reduced systemic vascular resistance and mean arterial pressure which decreased LVEDP and improved cardiac output.

The right ventricular systolic pressure is markedly higher with invasive assessment (73 mmHg) compared to noninvasive assessment (39 mmHg). This may be due to an inability to obtain an optimal continuous-wave Doppler signal or due to daily variation in right ventricular systolic pressure. Continuous daily invasive monitoring of right ventricular systolic pressure in PH type I patients has shown significant fluctuations [16], and we might expect similar fluctuations in patients with type II PH. Loading conditions can greatly affect the results of Doppler echocardiography, and exercise stress echocardiography may be quite helpful in identifying exercise-induced pulmonary hypertension and defining causes of exertional dyspnea.

## 4. Case Resolution

The patient was gradually initiated on a heart failure regimen consisting of carvedilol 25 mg twice daily, lisinopril 20 mg daily, furosemide 40 mg daily, and spironolactone 25 mg daily. The diltiazem was discontinued as it is a negative inotrope. The patient's blood pressure remained elevated, so hydralazine 50 mg three times daily and isosorbide mononitrate 30 mg daily was added to the regimen. Three-month follow-up demonstrated improved blood pressure control correlating with increased exercise tolerance.

## Figures and Tables

**Table 1:** Differential diagnosis for etiologies of heart failure with reduced ejection fraction (HFrEF).

Coronary artery disease
Hypertension
Diabetes
Familial cardiomyopathies
Tachycardia-induced cardiomyopathy
Infectious agents: bacterial, viral
Infiltrative disorders
Toxins
Nutritional deficiencies
Electrolyte disorders
Collagen vascular disorders
Endocrine and metabolic diseases
Peripartum cardiomyopathy
Obstructive sleep apnea
Idiopathic

Adapted from Mayo Board Review Textbook, Table 93.5, page 862.

**Table 2:** Physical exam findings of pulmonary hypertension.

Sign	Implication
Central cyanosis	Hypoxemia, right to left shunt
Clubbing	Congenital heart disease, pulmonary venopathy
Cardiac auscultation: murmurs, opening snap, gallop	Congenital or acquired heart or valvular disease
Rales, dullness, decreased breath sounds	Pulmonary congestion or effusion
Fine rales, accessory muscle use, wheezing, and prolonged expiration, productive cough	Pulmonary parenchymal disease
Obesity, kyphoscoliosis, enlarged tonsils	Disordered ventilation
Sclerodactyly, arthritis, rash	Connective tissue disorder
Peripheral venous insufficiency or obstruction	Venous thrombosis

Adapted from Mayo Board Review Table 79.3 (page 736).
